# Diversity and Functional Potential of Yeasts Inhabiting Honey Bee Drones

**DOI:** 10.3390/microorganisms13112614

**Published:** 2025-11-17

**Authors:** Vilija Lapinskaitė, Paulina Bartkutė, Juliana Lukša-Žebelovič, Živilė Strazdaitė-Žielienė, Elena Servienė

**Affiliations:** Laboratory of Genetics, State Scientific Research Institute Nature Research Centre, Akademijos Str. 2, LT-08412 Vilnius, Lithuania; vilija.lapinskaite@gamtc.lt (V.L.); pbartkute@gmail.com (P.B.); juliana.luksa@gamtc.lt (J.L.-Ž.); zivile.strazdaite-zieliene@gamtc.lt (Ž.S.-Ž.)

**Keywords:** *Apis mellifera*, honey bees, yeasts, biocontrol, autoaggregation, hydrophobicity, biofilms

## Abstract

The honey bee (*Apis mellifera* L.) is a eusocial insect widely known for its role in pollination and plant biodiversity. Diverse microorganisms, including both beneficial and pathogenic, colonize bees and play important roles in the overall hive health. Microorganisms with biocontrol properties are natural modulators of honey bee microflora. Since most studies have focused on the characterization of worker bee-associated microbes, there is a lack of information about the drones’ microbial environment. In this study, we identified cultivable yeasts from different stages of honey bee drones collected in Lithuania. Sealed larvae hosted the widest variety of yeasts. *Metschnikowia* species were detected across all developmental stages of drones. The assessment of functionality revealed that *M. pulcherrima* and *M. fructicola* exhibited the most pronounced biocontrol properties, accompanied by high levels of autoaggregation and hydrophobicity. *Starmerella apis* and *M. reukaufii* were distinguished by the highest autoaggregation capacity, exceeding 60%, and strong adherence to hydrocarbons. *Starmerella* genus yeasts demonstrated strong biofilm-forming ability. The novel information on the functionality of honey bee drone-inhabiting yeasts suggests their importance in maintaining the healthy microbiological environment of the hive. The isolated yeasts with beneficial traits may serve as candidates for future studies aimed at supporting honey bee health.

## 1. Introduction

Bees are considered the most crucial insect pollinators due to their significant contribution to plant reproduction and ecosystem stability [1]. Most crops worldwide rely on pollinators to grow and produce yields [2]. Among insect pollinators, the Western honey bees (*Apis mellifera* L.) hold global significance due to their vital role in pollinating crops, fruits, and wild plant species [3]. They provide an essential ecosystem service that supports both sustainable agricultural productivity and the preservation of natural ecosystems [3]. Bee products such as honey, pollen, bee bread, royal jelly, beeswax, and propolis have high economic importance and attractiveness to human health [3,4,5,6,7].

Honey bee colonies are highly organized social units comprising thousands of individuals. Each colony typically includes a few hundred males (drones), thousands of female workers, and a single reproductive queen [8]. The life cycle of the honey bee consists of four developmental stages: egg, larva, pupa, and adult [9]. After three days, the egg hatches into a white, legless, worm-like larva that is fed by worker bees. Nine days after the larva hatches, the honeycomb cells are sealed with a thin wax layer to allow the pupa to develop and then undergo a series of body changes until the emergence of an adult bee [10]. Worker bees perform diverse tasks essential for colony survival, including comb construction, food transportation, and hive defense, while drones are responsible for the fertilization of the queens [11].

Worker bees and drones host a complex microbiota comprising bacteria, yeasts, and other fungi that colonize various anatomical niches, including the gut, cuticle, and hive-stored resources [12]. The low microbial diversity has been observed in the gut of worker bees [12,13,14,15]. The bacterial community is dominated by core gut taxa such as *Gilliamella apicola*, *Snodgrassella alvi*, and *Lactobacillus* spp., which contribute to nutrient metabolism, pathogen defense, and immune modulation [15]. Yeasts are particularly abundant in the digestive tract and within hive materials such as pollen stores (bee bread) and fermented nectar [16]. A total of seventeen distinct yeast genera were identified in the guts of honey bees, regardless of hive origin. The genus *Hanseniaspora* was the most prevalent, comprising four different species, followed by *Pichia* and *Rhodotorula*, each represented by three species [16]. A study on three different bee subspecies (*Apis mellifera ligustica*, *Apis mellifera jemenitica*, and *Apis florea*) revealed that *Starmerella* spp. was the dominant cultivable fungal microorganism, followed by *Hanseniaspora* spp., *Aspergillus* spp., and *Naganishia* spp. Other species from the *Aureobasidium*, *Moniliella*, *Candida*, and *Penicillium* genera were detected in lower proportions [17,18].

Honey bees suffer from microbial and viral infections, and to combat them, they activate innate immune reactions. Drone and worker larvae respond to bacterial infection by producing antimicrobial peptides, while pupae are incapable of activating humoral or cellular immune responses [9]. Microorganisms colonizing honey bees stimulate the host immune system, and by producing biocontrol agents, directly act against pathogens [16,19]. Among the microorganisms, certain yeast species play a beneficial role through biocontrol mechanisms, producing antimicrobial metabolites and toxins that inhibit pathogenic fungi and bacteria [20]. For instance, *Metschnikowia pulcherrima* synthesizes pulcherriminic acid, which chelates iron and suppresses the growth of competing microbes, including honey bee pathogens such as *Ascosphaera apis* [21]. Yeast-derived volatile compounds and secondary metabolites can also modulate the hive microbiome and suppress spoilage organisms [22]. The specific environment in the hive and honey bees could act as a reservoir for functional yeasts [23]. Yeasts from the genera *Starmerella* and *Metchnikowia* exhibit probiotic traits, including hydrophobicity and autoaggregation. These traits enhance their ability to adhere to host epithelial surfaces, compete effectively in the gut environment, and protect against harmful pathogens [16,17,18,23]. However, some yeast species, such as opportunistic *Wickerhamomyces* spp., are associated with pathogenicity in bees, particularly under stress conditions or in colonies exposed to antibiotics [24]. Pathogenic yeasts may cause mycoses, disrupt the gut microbial balance, and contribute to malnutrition or weakened immunity, especially in drones and older worker bees. The dual nature of bee-associated yeasts underscores their importance as both beneficial symbionts and potential pathogens within the hive ecosystem [25,26].

Honey bee drones, although not directly involved in honey production, play a crucial role in maintaining the overall microbiological environment of the hive [27]. They can transmit microorganisms to the queen during the fertilization process and carry microorganisms when moving from one hive to another [28,29]. Despite numerous studies investigating the worker honey bee-inhabiting microorganisms [26,30,31], much less is known about the microbes associated with honey bee drones [28,32]. Some studies reveal that the gut bacterial composition changes during the development of worker bees [33,34]; however, to the best of our knowledge, there are no studies on the microflora of drones at different developmental stages. Furthermore, there is a lack of information on the yeast communities distributed throughout the entire body of drones. Recently, particular attention has been paid to natural means of enhancing honey bee health, including antimicrobial properties-possessing microorganisms [35,36]. Biocontrol yeasts could be very attractive in this respect, by synthesizing antimicrobial metabolites that protect against the spread of pathogens and diseases, thus may play a crucial role in the regulation of a favorable microflora to bee health. Therefore, there remains a high demand for research on beneficial features-possessing honey bee-associated yeasts. In this study, we aimed to isolate and identify cultivable yeasts associated with different developmental stages of honey bee drones, explore their biocidal and probiotic properties that may have positive contributions to honey bee health.

## 2. Materials and Methods

### 2.1. Collection of Honey Bee Drones and Ethics Statement

**Figure 1:**
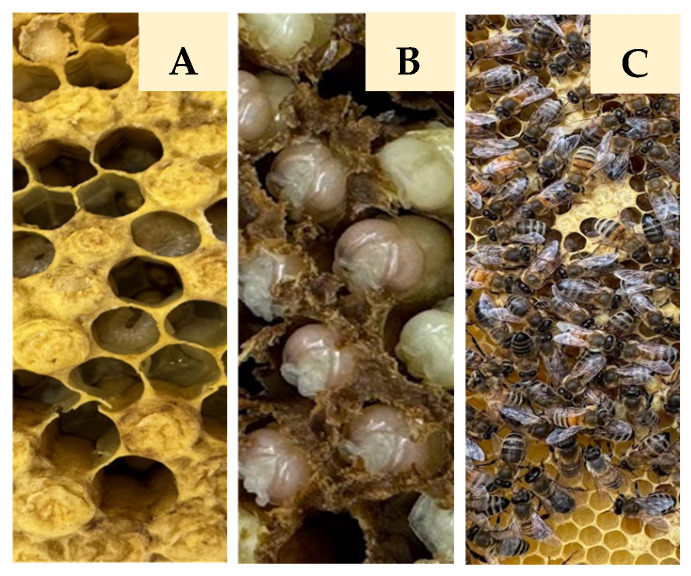
Sampling of honey bee drones at different developmental stages ((**A**)—unsealed and sealed larvae (ULD/SLD); (**B**)—pupae (PD); (**C**)—adults (AD)).

Honey bee (*Apis mellifera* L.) drones were aseptically collected six times between May and July 2024 at the ecological apiary “Medaus namai” located in Antazavė, Zarasai region, Lithuania (GPS coordinates: 55.815922, 25.918649). Six honeycombs (15 × 45 cm) containing drone brood were placed in sterile bags. The samples were transported to the laboratory and processed within 2–3 h for further analysis. Drones at different developmental stages (unsealed larvae, sealed larvae, pupae, and adults) (Figure 1) were aseptically selected from the combs and used for isolation of cultivable yeasts.

The private owner authorized the collection of honey bee drones. Endangered or protected species were not involved in this study, and no specific permissions were required.

### 2.2. Cultivable Microorganisms Isolation

For the isolation of cultivable yeasts, 10 g of drones from each developmental stage were aseptically removed from the combs and homogenized for 3 min using sterile mortar and pestle in 30 mL of MD medium (2% glucose, 1% (NH_4_)_2_SO_4_, 0.09% KH_2_PO_4_, 0.05% MgSO_4_, 0.023% K_2_HPO_4_, 0.01% NaCl, 0.01% CaCl_2_). The homogenate was filtered through a 1.5 mm wire mesh to remove drone remnants and then centrifuged at 800 rpm for 10 min. The supernatants were serially diluted in 0.9% NaCl, and aliquots were plated in triplicate on Petri dishes, containing either Plate count agar (PCA) (1% glucose, 2.5% yeast extract, 5% tryptone, 1.5% agar, supplemented with 50 g mL^−1^ chloramphenicol) or yeast extract-peptone-dextrose (YPD) media (1% yeast extract, 2% peptone, 2% glucose, 2% agar, supplemented with 50 g mL^−1^ chloramphenicol). YPD and PCA plates were incubated at 25 °C for 2 days. Colony-forming units (CFUs) representing fungal microorganisms were counted, and results were expressed as log CFU/g honey bee drones.

### 2.3. Identification of Isolated Yeasts

Morphologically distinct microorganisms were streak-plated from single colonies onto YPD and PCA medium plates for further analysis. The structure, size, and colors of the colonies were noted, and cells were analyzed under the light microscope (Leica DM750, Wetzlar, Germany), and micrographs were recorded using the digital camera (Leica ICC50 HD, Wetzlar, Germany).

For molecular identification, 10 mL of each culture was grown overnight in YPD medium and centrifuged at 5000× *g* for 5 min at room temperature. Supernatant was removed, and microbial DNA was extracted from the obtained 70–100 mg pellet using the manufacturer’s protocol for the Yeast DNA Extraction Kit (Thermo Fisher Scientific Baltics, Vilnius, Lithuania). Extracted DNA was subjected to PCR analysis. PCR amplification of the region between the 18S and 28S rRNA genes was performed using ITS1 (5′-TCCGTAGGTGAACCTGCGG-3′) and ITS4 (5′-TCCTCCGCTTATTGATATGC-3′) primers. For identification and phylogenetic analysis of yeasts, the D1/D2 region of 26S rDNA was amplified using NL1 (5′-GCATATCAATAAGCGGAGGAAAAG-3′) and NL4 (5′-GGTCCGTGTTTCAAGACGG-3′) primers.

The reaction mixtures (50 µL) were composed of 5 μL Dream Taq buffer, 5 μL of 2 mM dNTP mix, 1 μL of each primer (10 μmol/L), 2.5 units of DreamTaq DNA polymerase, 4 μL of lysed cells, and sterile distilled water to the final volume. For ITS region amplification, ITS1 and ITS4 primers were used; the PCR conditions were as follows: initial denaturation at 94 °C for 5 min; 25 cycles of 94 °C for 1 min, 53 °C for 1 min 30 s, 72 °C for 2 min; a final elongation at 72 °C for 10 min. For D1/D2 region amplification, PCR conditions were the same, except the annealing temperature was 52 °C. The size of all PCR products was analyzed by 1% agarose gel electrophoresis compared to GeneRuler DNA Ladder Mix.

ITS PCR products were subjected to RFLP analysis for preliminary species identification. PCR products were digested with FastDigest HinfI and HhaI restriction enzymes, and restriction profiles were checked by 2% agarose gel electrophoresis. Selected D1/D2 PCR products were purified using the GeneJet PCR purification kit (Thermo Fisher Scientific Baltics, Vilnius, Lithuania) according to the manufacturer’s instructions and sequenced using NL1/NL4 primers at BaseClear (Leiden, The Netherlands). Generated sequences were analyzed using Chromas (version 2.6.6) software and compared to reference sequences using BLAST + 2.17.0 version (https://blast.ncbi.nlm.nih.gov/Blast.cgi?PROGRAM=blastn&PAGE_TYPE=BlastSearch&LINK_LOC=blasthome, accessed on 12 September 2025). Newly obtained sequences were deposited in the National Center for Biotechnology Information (NCBI) GenBank under accession numbers PX353702–PX353734. Sequences showing >97% were assigned to the corresponding genus and/or species. All chemicals for PCR and restriction reactions were manufactured by Thermo Fisher Scientific Baltics, Vilnius, Lithuania, while the primers were obtained from Metabion, Germany.

### 2.4. Phylogenetic Analysis

Phylogenetic analysis was conducted using 26S rRNA gene sequences (D1/D2 region) obtained from honey bee drones at different development stages (*n* = 32), along with reference sequences retrieved from the NCBI GenBank database (*n* = 19). Multiple sequence alignment was carried out using the MUSCLE (implemented in MEGA12, version 12.0.11) and manually adjusted using the MEGA alignment editor where required. A phylogenetic tree was constructed in MEGA12 using the Maximum Likelihood (ML) method. Evolutionary distances were estimated by applying the General Time-Reversible model with gamma distribution and a proportion of invariant sites (GTR + G + I). The robustness of the inferred tree topology was assessed through bootstrap analysis with 1000 replicates. Nineteen representative reference sequences from the same species and targeting the same genomic region as our sequences were retrieved from the NCBI GenBank database to ensure comprehensive representation of related taxa [37].

### 2.5. In Vitro Evaluation of Antifungal Activity

For the detection of killing phenotype, the tested yeast strain was spotted on the MBA agar plates (0.5% yeast extract, 0.5% peptone, 2% dextrose, 2% agar, 0.002% methylene blue, pH 4.8) seeded with a lawn (2 × 10^6^ cells/plate) of different yeast species (*Rhodotorula graminis*, *R. mucilaginosa*, *Candida albicans*, *Nakaseomyces glabratus*, *Sporobolomyces roseus*, *Cryptococcus wieringae*, *Aureobasidium pullulans*, *Starmerella magnoliae*) selected from the collection of microorganisms maintained at the culture collection of the Laboratory of Genetics, State Scientific Research Institute Nature Research Centre, Lithuania. The plates were incubated at 25 °C for 2–3 days, and the antifungal activity was evaluated based on the appearance of clear zones of growth inhibition surrounding the spotted cells.

### 2.6. Determination of Yeast Antibacterial Activity

Antibacterial activity of isolated yeast strains was tested against the four Gram-positive (*Listeria innocua* CECT 910T (University of Lisbon, Portugal), *Bacillus subtilis* ATCC 6633, *Streptococcus pyogenes* ATCC 19615, *Staphylococcus aureus* ATCC 29213 (Vilnius University, Lithuania)) and four Gram-negative (*Escherichia coli* BL21 [F-dcm ompT hsdS(rB-mB-) gall (DE3)] (Thermo Fisher Scientific Baltics, Vilnius, Lithuania), *Klebsiella pneumoniae* KV-3, *Pseudomonas aeruginosa* ATCC 27853 (Vilnius University, Lithuania), *Salmonella typhimurium* LT2 (Vilnius Tech University, Lithuania)) bacteria. Each bacterial strain was grown overnight in Luria–Bertani (LB) medium (0.5% yeast extract, 1% peptone, 1% NaCl) at 37 °C and seeded with a lawn (2 × 10^7^ cells/plate) in LB agar medium adjusted to pH 5.6 and supplemented with 2% glucose. The yeast strains to be tested were grown in YPD medium for 24 h at 30 °C, and 5 µL of the overnight yeast culture (OD 1) was spotted on top of the bacteria-seeded plates. The plates were incubated at 30 °C for 1–2 days. The presence of an inhibitory zone around the spotted cells was assessed as an antibacterial activity.

### 2.7. Determination of Yeast Autoaggregation Properties

Autoaggregation ability was determined as described [38], with minor modifications, using the autoaggregation percentage. More specifically, yeast cultures were grown overnight in flasks with YPD medium, yeast cells were collected by centrifuging at 5000× *g* for 10 min, washed twice with 1X phosphate-buffered saline (PBS—0.8% NaCl, 0.02% KCl, 0.29% Na_2_HPO_4_ × 12H_2_O, 0.0244% KH_2_PO_4_, pH 7.2) solution to remove the residual growth medium, and resuspended in the same buffer. The optical density (OD) of the resulting solution was adjusted to 0.25 ± 0.05 at 600 nm. The suspension was vortexed for 10 s and incubated at 37 °C for 24 h. After 3 and 24 h of incubation at 37 °C, 1 mL of suspension was taken, and the OD was measured at 600 nm. The percentage of autoaggregated yeasts was calculated using the following formula:

Autoaggregation (%) = 1 − A_t_/A_0_ × 100;

A_t_—OD_600nm_ at different time points;

A_0_—OD_600nm_ at 0 h.

### 2.8. Yeast Adhesion to Hydrocarbons

The hydrophobicity of yeasts was estimated according to [38] with slight modification. Yeast cultures were grown overnight in YPD medium. Yeast cells were centrifuged at 5000× *g* for 10 min and washed twice with 1X PBS (pH 7.2) and resuspended in the same buffer. The cell suspension was adjusted to an OD of 0.6 at 600 nm, and 1 mL of each suspension was added to 0.2 mL of hydrocarbon (xylene or hexane) and vortexed for 120 s. The two phases were allowed to separate for 1 h at 37 °C. The aqueous phase was carefully removed, and the OD_600_ was measured. The decrease in the absorbance of the aqueous phase was taken as a measure of the cell surface hydrophobicity (%), which was calculated using the formula:

Hydrophobicity (%) = (OD_0_ − OD/OD_0_) × 100;

OD_0_—before extraction with hydrocarbon;

OD—after extraction with hydrocarbon.

### 2.9. Biofilm Formation

For biofilm quantification, the microtiter plate crystal violet assay was used as described [39] with minor modifications. Overnight-grown yeast cultures were diluted in YPD medium to an OD of 0.2 at 600 nm, and 200 µL of yeast suspension was placed into a sterile 96-well plate (TPP 96-well Culture plate flat bottom, Sigma-Aldrich, Buchs, Switzerland). The yeast cells were incubated at 30 °C for 72 h. As a negative control, not forming biofilms, the *S. cerevisiae flo11* deletion strain (Thermo Scientific Molecular Biology, Lafayette, CO, USA) was used [40], while YPD medium served as a blank control. After the incubation, medium from biofilm-containing wells was removed, and wells were gently washed twice with 200 μL of 1X PBS (pH 7.2) to remove planktonic cells. Then the biofilms were stained for 10 min with 200 μL of crystal violet solution (0.1%) at room temperature, washed twice with PBS, and air dried. Finally, 200 μL of 33% acetic acid was added to every well and incubated at 150 rpm for 30 min at room temperature. The absorbance of 570 nm was measured by a microplate reader (Tecan Infinite M PLEX, Grödig, Austria) operated with Tecan i-Control software (version 3.9.1.0). The assessment of biofilm-forming ability was conducted using the methodology described by [41,42]. YPD medium was chosen as a negative control for the calculations. The average for all tested strains and the negative control was calculated, as all tests were performed in triplicate. The cut-off value (ODc) was defined as: ODc = average OD of negative control + (3 × standard deviation (SD) of negative control). Final OD value of all tested strains was expressed as: OD = average OD of a strain − ODc. The results are classified as presented in Table 1.

### 2.10. Statistical Analysis

All experiments were performed in triplicate, and results are expressed as mean ± standard deviation. Statistical analysis was carried out using Microsoft Office Excel 2016. Biofilm formation data were analyzed in R (version 4.4.3; [43]) using RStudio (version 2024.12.1; Posit Software, PBC). Biofilm formation measurements were compared using the Kruskal–Wallis rank-sum test, followed by Dunn’s multiple comparisons test with Bonferroni correction to identify significant pairwise differences. Analyses were performed with the FSA (version 0.10.0; [44]) and the dunn.test (version 1.3.6; [45]) packages, with *p* < 0.05 considered statistically significant.

## 3. Results and Discussion

### 3.1. Cultivable Yeasts Associated with Honey Bee Drones at Different Development Stages and Phylogenetic Characterization

In the present study, we analyzed the composition of cultivable yeasts using whole-body homogenization of honey bee drones at different developmental stages. Thus, the recovered strains represent the overall cultivable yeast community of drones. A viable count assay was performed by plating the samples on PCA and YPD plates supplemented with chloramphenicol (Table 2). Different media were selected to ensure comprehensive coverage of cultivable microorganisms, particularly yeasts. The highest viable counts were determined in the adult samples regardless of the media used: on PCA plates, 5.1 ± 0.46 log CFU/g, and on YPD, 4.6 ± 0.35 log CFU/g. Meanwhile, when examining samples from the other developmental stages, the total aerobic counts (TAC) varied depending on the medium used. When using PCA, the lowest number of viable counts was observed in sealed larvae (2.11 ± 0.37 log CFU/g), while on YPD media—in unsealed larvae (1.52 ± 0.79 log CFU/g). Pupae exhibited a moderate amount of CFU on both media (2.26 ± 0.26 log CFU/g on YPD and 3.81 ± 0.84 log CFU/g on PCA). In most cases, except for sealed larvae, more viable counts were detected on PCA medium, which is general for a broad range of microorganisms compared to yeast-specific YPD medium. Previous studies based on cultivation-dependent approaches have shown that in adult bees from *A. mellifera ligustica*, *A. mellifera jemenitica*, and *A. florea* species, the number of fungal cells per mg of gut tissue ranged from 2.7 × 10^2^ to 1 × 10^4^, depending on the region tested [17]. The slightly different yeast counts observed in our study may therefore reflect variations in sampling approach and microbial localization (gut vs. entire body) between drones and workers, rather than actual variances in overall yeast abundance.

**Figure 2:**
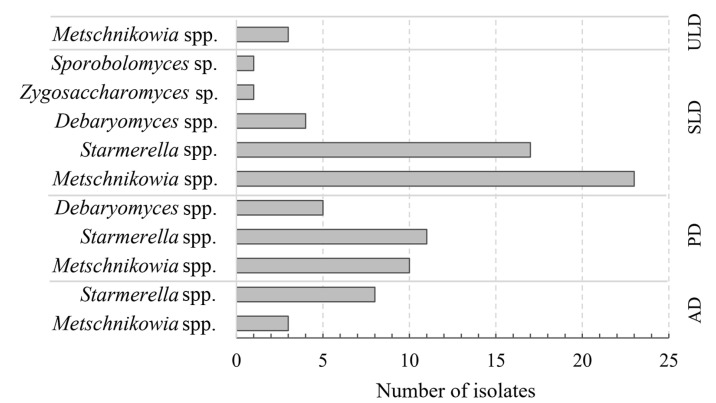
Presence of yeast species on different stages of honey bee drones (ULD—unsealed larvae; SLD—sealed larvae; PD—pupae; AD—adults).

By applying cultivation techniques, 86 yeast isolates were distinguished from different developmental stages of honey bee drones. Yeast strains were subjected to morphological colony analysis, bright-field microscopy of the cells, and the following fingerprinting of PCR-amplified ITS markers (Table S1). It was determined that the highest number of yeast isolates (46) differing by morphological features and RFLP profiles were detected in sealed larvae honey bee drone samples. *Starmerella* and *Metschnikowia* genus yeasts were the most prevalent (30 and 41%, respectively). There were half as many yeast isolates observed from the pupae stage (26), followed by adults (11 isolates) and unsealed larva samples (3) (Figure 2).

For further analysis, selected strains representing all developmental stages of drones were sequenced and used for species-level identification, with the resulting sequences deposited in the NCBI database (Table 3).

*Metschnikowia* genus yeasts were found in all developmental stages of honey bee drones. Sealed larvae, despite low abundance, hosted the widest variety of yeasts, including *M. pulcherrima*, *M. reukaufii*, *M. fructicola*, *S. magnoliae*, *S. sorbosivorans*, *S. apis*, *S. apicola*, *S. bombi*, *D. hansenii*, *Z. rouxii*, and *Sporobolomyces* sp. In the pupal stage, the most common yeasts were *M. pulcherrima*, *S. bombi*, *S. apicola*, *S. magnoliae*, and *D. hansenii*. Adult drones were associated with *M. pulcherrima* and *S. magnoliae*. Only *M. pulcherrima* was found on the unsealed larvae (Table 3). Our findings revealed that yeast abundance and diversity vary across the developmental stages of honey bee drones.

A Maximum Likelihood tree was generated based on sequences of the D1/D2 regions of identified yeast species (Figure S1). Clades supported by the highest bootstrap values in the phylogenetic analysis showed a strong correspondence with the respective yeast species. Among the isolates, it was possible to discern similarity between yeasts of the same species representing different stages of development of honey bee drones. In the phylogenetic tree, *M. pulcherrima* PD2 (isolated from pupae) was grouped with *M. pulcherrima* AD1 (isolated from adults), and *S. apicola* PD1 (from pupae) was placed in a cluster with *S. apicola* SLD14 (from sealed larvae). While other *M. pulcherrima* strains, such as SLD20 and SLD25, ULD4 and ULD5, and *S. bombi* PD6 and PD7, formed separate groups, showing higher similarity between isolates from the same honey bee drone developmental stage. *S. magnoliae* and *D. hansenii* isolates formed sister groups regardless of drone developmental stage.

Different factors, such as biogeography, seasonal climate changes, different floral sources, microbial environment of hive and stored provisions, may affect the composition of honey bee-associated microbial communities [46]. The analysis of the cultivable yeast community present in honey bee workers collected from an apiary in Italy demonstrated that the *Meyerozyma*, *Hanseniaspora*, and *Pichia* genera were the most prevalent [16]. Similar to our study, *Metschnikowia* spp., *Debaryomyces* spp., *Starmerella* spp., and *Sporobolomyces* spp. were also detected in the gut of bee workers, but at reduced levels (ranging from 2% to 4%). *Starmerella* genus yeasts prevailed in stingless bees (31%) [18]. Honey bee gut microbiota is affected by diet, climate, and hive member caste; however, it is more stable than that on the body surface [33]. We analyzed yeast isolated from the entire body of honey bee drones, so their diversity may also be dependent on the hive environment. In honey bee products (bee pollen, bee bread, propolis), the majority of identified yeasts belonged to *Metschnikowia* spp., *Starmerella* spp., *Filobasidium* spp., *Meyerozyma* spp., and *Debaryomyces* spp. (from 7% to 20%), while *Zygosaccharomyces* spp. constituted only 2% [16]. Identified yeast communities, such as *Starmerella*, *Metschnikowia*, and *Zygosaccharomyces*, which dominated different stages of bee bread maturation, frequently occurred in nectar, pollen, and provisions [47,48]. Numerous yeasts found in bee products are involved in fermentation processes, and they are similar to those observed in either worker bees or drones. *Metschnikowia*, *Starmerella*, *Hanseniaspora*, *Candida*, *Zygosaccharomyces*, etc., have been frequently isolated from flowers and their visiting bees, thus suggesting that they are acquired by bee workers during foraging and transmitted further to hive members [17,46]. The phylogenetic similarity among isolates from different developmental stages points to the potential transfer of yeasts from the extra-hive environment to drone larvae by nurse bees during the feeding process, and further microbial exchange between pupae and mature drones through the shared environment [33,46]. The distribution and abundance of various yeast species across the developmental stages of honey bee drones may depend on the food received, social interactions between hive members, and the metabolic conditions of the gut, which change during development [33]. Different limitations, such as formulations of culturing media, incubation conditions, interactions and competitions between various microorganisms, may affect the culturable yeast recovery. For a comprehensive analysis of yeast communities, culture-based and culture-independent approaches should be combined.

### 3.2. Biocontrol Features of Honey Bee Drones-Inhabiting Yeasts

**Figure 3:**
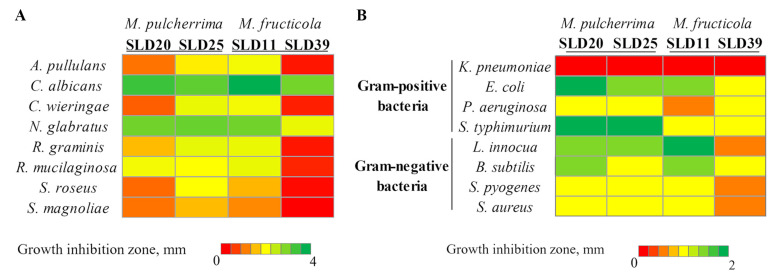
Antimicrobial activity of *Metschnikowia* genus yeast strains against fungal microorganisms (**A**) and bacteria (**B**).

Yeast strains isolated from sealed larvae were selected for biocontrol analysis because this group exhibited the highest species diversity and represented all dominant genera detected across drone developmental stages. The antifungal activity against *Aureobasidium*, *Candida*, *Cryptococcus*, *Nakaseomyces*, *Rhodotorula*, *Starmerella*, and *Sporobolomyces* yeasts was evaluated (Figure 3). Some of these species may be attributed to potential human and plant pathogens [49,50,51,52,53,54,55].

The extent of inhibition varied depending on both the tested and target microorganisms. Among all analyzed strains, *M. pulcherrima* SLD20 and SLD25, as well as *M. fructicola* SLD11 and SLD39, demonstrated antifungal activity (Figure 3A). Other yeasts (*M. reukaufii*, *S. magnoliae*, *S. apis*, *S. apicola*, *S. bombi*, *S. sorbosivorans*, *Z. rouxii*, *D. hansenii*, and *Sporobolomyces* sp.) have not shown any detectable antifungal activity. *M. pulcherrima* SLD25 and *M. fructicola* SLD11 strains possessed the strongest antifungal properties by forming inhibition zones from 1.5 to 3.8 mm, depending on the target microorganism. *M. pulcherrima* SLD20 and *M. fructicola* SLD39 isolates demonstrated slightly lower activity. The strongest inhibitory effects of *Metschnikowia* strains were observed against *C. albicans* (the diameter of inhibition zones was in the range of 2.9–3.8 mm) and *N. glabratus* (lysis zones from 2 to 3 mm) yeasts. *S. magnoliae* and *S. roseus* were among the least affected species (lysis zones 0.3–1.5 mm and 0.4–1.9 mm, respectively).

The antibacterial efficacy of isolated from sealed larvae yeast strains was evaluated against a model Gram-positive (*L. innocua*, *B. subtilis*, *S. pyogenes*, *S. aureus*) and Gram-negative (*K. pneumoniae*, *E. coli*, *P. aeruginosa*, *S. typhimurium*) bacteria. Among the tested 17 yeast strains, only *M. pulcherrima* SLD20 and SLD25 and *M. fructicola* SLD11 and SLD39 strains demonstrated broad-spectrum antibacterial activity (Figure 3B). Other tested yeasts (*S. magnoliae*, *M. reukaufii*, *S. sorbosivorans*, *S. apis*, *S. apicola*, *S. bombi*, *D. hansenii*, *Z. rouxii*, and *Sporobolomyces* sp.) did not affect the tested bacterial strains. *M. pulcherrima* SLD20 and SLD25 strains exhibited similar antibacterial activity, with the strongest one against *S. typhimurium* (inhibition zone diameter 2 mm), *E. coli* (zone diameter 2 and 1.5 mm, respectively), and *L. innocua* (zone diameter about 1.5 mm). The antibacterial activity of the *M. fructicola* strains differed to a greater extent. *M. fructicola* SLD11 strain demonstrated the greatest activity against *L. innocua* and *E. coli* (inhibition zone diameters 2 and 1.5 mm, respectively), while the activity of SLD39 strain against mentioned above bacterial species was lower. All tested *Metschnikowia* strains showed low antibacterial activity against *P. aeruginosa*, *S. pyogenes*, and *S. aureus*, but none of the strains were able to act against *K. pneumoniae*.

The biocidal activity of yeasts may be caused by the production of antimicrobial agents, such as killer toxins, volatile organic compounds, or other secondary metabolites [56,57]. Several mechanisms may be involved in the execution of biocontrol, and each of them is yeast species-dependent [56,58]. One of the most potent biocontrol mechanisms observed in yeasts is based on the ability to produce proteinaceous killer toxins that inhibit the growth of other microorganisms [59]. *M. pulcherrima* yeasts were able to synthesize toxin, demonstrating killing activity at a broad pH spectrum [56,59]. The inhibitory mechanism of *Metschnikowia* yeasts may also rely on the pH-dependent production of pulcherrimic acid, which chelates iron to form the pigment pulcherrimin, inhibiting the growth of susceptible microorganisms by sequestering iron [19,60]. *M. pulcherrima* and *M. fructicola* are known for their ability to inhibit the growth of undesirable yeasts (such as *Aureobasidium pullulans*, *Rhodotorula glutinis*, *Sporobolomyces roseus*, etc.) [61,62], and bacteria (such as *Salmonella enterica*, *Staphylococcus aureus*, and *Escherichia coli* [63], thus demonstrating high biocontrol potential. In our study, *Metschnikowia* yeast strains exhibited broad-spectrum antimicrobial properties, thus may be beneficial for maintaining the health of bees and the entire hive.

### 3.3. Evaluation of Probiotic Traits

#### 3.3.1. Variation in Autoaggregation Capacity Across Strains

Autoaggregation of microorganisms is essential for their adhesion to epithelial cells and mucosal surfaces, which is a critical step for effective colonization of the gastrointestinal tract [64,65]. Also, autoaggregation is considered the initial step in the adhesion process, enabling probiotic microorganisms to form a protective barrier that can inhibit the attachment of undesirable or pathogenic microorganisms [66].

**Figure 4:**
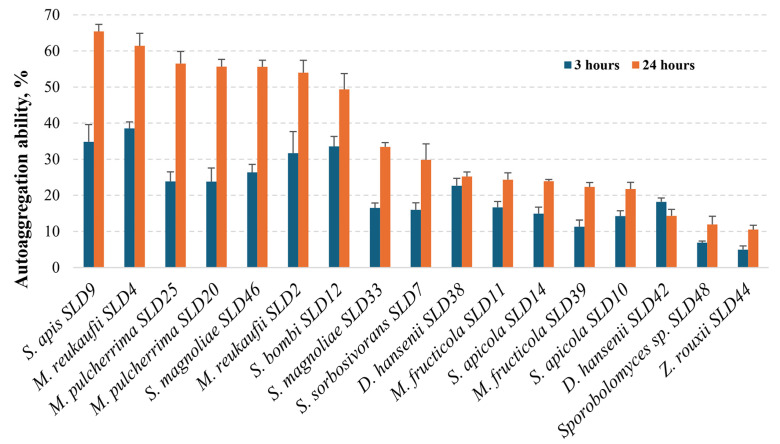
Autoaggregation ability of yeast isolates from honey bee drone larvae.

In our study, the ability of the tested microorganisms to self-aggregate was analyzed after 3 and 24 h of incubation at 37 °C (Figure 4). Comparison of the mean percentage auto-aggregation values after 3 h of incubation showed that the highest 30% exceeding values were detected for the yeast species *M. reukaufii* (SLD2 and SLD4) (31.7% and 38.5%), *S. apis* SLD9 (34.8%), and *S. bombi* SLD12 (33.5%). Both *S. magnoliae* strains (SLD33 and SLD46) had slightly lower capacities for self-aggregation (16.5% and 26.3%, respectively), similar to *M. pulcherrima* SLD20 and SLD25 strains (both 23.8%). After 24 h of incubation, an overall increase in autoaggregation was observed. *S. apis* SLD9 and *M. reukaufii* (SLD4) showed the highest capacity to autoaggregate (more than 60%). The *M. pulcherrima* SLD20 and SLD25, *S. magnoliae* SLD33 and SLD46, *S. bombi* SLD12, *S. sorbosivorans* SLD7, and *M. reukaufii* SLD2 strains showed moderate autoaggregation (between 30% and 60%). The remaining strains (e.g., *D. hansenii* SLD38, *M. fructicola* SLD11 and SLD39, *S. apicola* SLD10 and SLD14, *Sporobolomyces* sp. SLD48, and *Z. rouxii* SLD44) exhibited low autoaggregation (<30% after 24 h).

Our study is in line with others that observed time-wise increasing autoaggregation rates for numerous yeast species, pointing towards *M. pulcherrima* strains with high self-aggregation properties [67]. Wildly recognized probiotic yeasts belonging to the *Saccharomyces boulardii* species were differentiated by high autoaggregation capacity (up to 81%) [68]. After isolating yeasts from bee bread and pollen, an autoaggregation study showed that the results for *S. magnoliae* varied between 35 and 37%. Other yeasts of the genus *Starmerella*, which were not identified to species level, showed varying autoaggregation ability, ranging from 20 to 75% [23]. Our results suggest that the strains from *S. apis*, *M. reukaufii*, *M. pulcherrima*, and *S. bombi*, with a high, increasing over time autoaggregation potential, could be considered as possessing probiotic aptitude.

#### 3.3.2. Hydrophobic Interaction with Hydrocarbons

The adhesive capacity of probiotics is commonly evaluated through a combination of surface hydrophobicity and autoaggregation assays [69]. The adherence to hydrocarbons assay is a widely used method for assessing the cell surface hydrophobicity of probiotics by evaluating their affinity to solvents [70].

**Figure 5:**
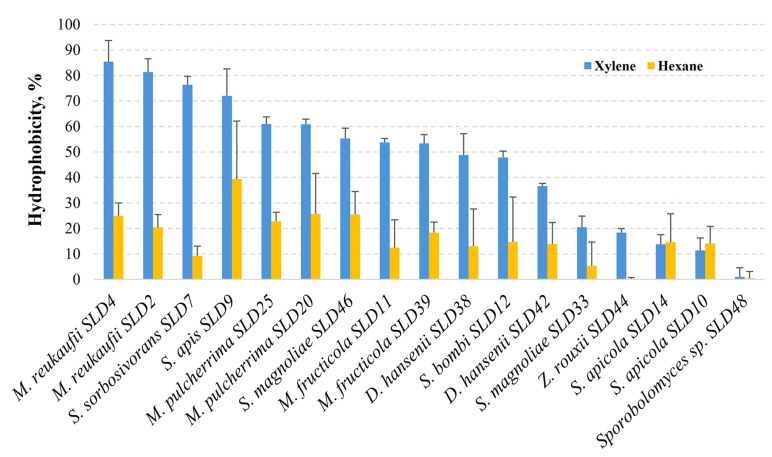
Hydrophobicity ability of yeast isolates from honey bee drone larvae.

Hexane and xylene were used as organic solvents for the hydrophobicity test. The results of the hydrophobicity test are shown in Figure 5. When xylene was used as a hydrocarbon, *M. reukaufii* SLD4 and SLD2, *S. sorbosivorans* SLD7, *S. apis* SLD9, *M. pulcherrima* SLD25, and SLD20 strains showed a high degree of hydrophobicity of over 60%. The species with the lowest hydrophobicity (less than 30%) were *S. apicola* (SLD14 and SLD10) (13.8% and 11.3%, respectively) and *Z. rouxii* SLD44 (18.4%), while no hydrophobicity characteristics were observed in *Sporobolomyces* sp. SLD48. The other tested yeasts showed moderate (30% to 60%) hydrophobicity properties. Among the yeasts with moderate hydrophobicity, *M. fructicola* (SLD11 and SLD39) (53.4% and 53.7%, respectively), *S. magnoliae* SLD46 (55.3%), and *S. bombi* SLD12 (47.9%) isolates were the most significant.

When hydrocarbon was changed to hexane, *S. apis* SLD9 showed the strongest hydrophobicity (39.4%), followed by *S. magnoliae* SLD46 (25.5%), *M. pulcherima* (SLD25 and SLD20) (25.7% and 22.8%, respectively), and *M. reukaufii* (SLD4 and SLD2) (25% and 20.4%, respectively). These hydrophobicity results mainly confirm those obtained in the xylene test. The lowest hydrophobicity was observed for *S. sorbosivorans* SLD7 (9.2%) and *Z. rouxii* SLD44 (0.1%). As with xylene, negligible (<1%) hydrophobicity properties were observed in *Sporobolomyces* sp. SLD48.

According to hydrophobicity studies, most microorganisms can interact with organic solvents, but certain species clearly have high hydrophobicity indices [71]. Yeast strains isolated from honey bee bread and pollen possessed important probiotic features, including adhesive capacities. When hexadecane was used as a solvent, *S. magnoliae* hydrophobicity varied from 6.21% to 50.71%; another *Starmerella* genus strain’s ability to adhere to hydrocarbons also differed significantly (from 5.32% to 59.86%) [23]. After conducting a hydrophobicity test with probiotic yeast *Saccharomyces boulardii*, it was found that when two hydrocarbons (hexane and xylene) were used, their hydrophobicity reached 32.84% and 34.73%, respectively [72]. In our study, microorganisms such as *S. apis*, *M. reukaufii* showed the highest hydrophobicity results; *M*. *pulcherrima*, *M. fructicola*, *S. magnoliae*, and *S. bombi* showed moderate to high hydrophobicity. The results of the hydrophobicity test provide insight into the surface properties of the microorganisms studied, which are associated with their ability to adhere to intestinal epithelial cells [73].

Hydrophobicity and autoaggregation are closely related properties; thus, microorganisms possessing higher hydrophobicity demonstrate stronger autoaggregation ability [70]. This correlation was also observed in our study, where *S. apis*, *M. reukaufii*, and *M*. *pulcherrima* strains were distinguished by both characteristics, which could be important for pathogen exclusion and maintaining healthy honey bees.

#### 3.3.3. Determination of Biofilm Formation Potential

The ability of yeast to form biofilms is a significant factor in competing for space and may be an important biocontrol solution in preventing pathogenic microorganisms and protecting against infection [57]. Yeast strains exhibited varying capacities for biofilm formation, highlighting species- and strain-specific differences in this important phenotypic trait. The ability of yeast strains to form biofilms on the plastic surface was evaluated after 72 h (Table 4). The classification of biofilm values was presented in Table 1. *S. cerevisiae flo11* was selected as a strain that cannot form biofilms. In the weak biofilm-forming range (OD_570_ from 0.49 to 0.98) were *S. cerevisiae flo11*, *D. hansenii* SLD38, and *Z. rouxii* SLD44 strains. Moderate biofilm (OD_570_ from 0.98 to 1.96) formation, according to calculations, was primarily demonstrated by species belonging to the *Metschnikowia* genus—namely, *M. fructicola* SLD11 and SLD39, *M. reukaufii* SLD2 and SLD4, and *M. pulcherrima* SLD20 and SLD25, as well as by *Sporobolomyces* sp. SLD48. The strongest ability to form biofilms was demonstrated by all *Starmerella* genus strains (*S. magnoliae* SLD33 and SLD46, *S. apicola* SLD10 and SLD14, *S. sorbosivorans* SLD7 and SLD24, *S. bombi* SLD12, *S. apis* SLD9 strains), and *D. hansenii* SLD42 isolate.

Biofilm formation is considered an important mode of action in biocontrol yeasts. Biofilm formation has been implicated in the protective and biocontrol activities of *M. pulcherrima* [61]. Some *Starmerella* genus strains isolated from the honey bee environment demonstrated a high capacity in forming biofilms and were also distinguished by increased autoaggregation and hydrophobicity. Even more, these probiotic features-possessing strains display antimicrobial activity against pathogenic bacteria and fungi [23]. The robust biofilm development in some yeast strains could be linked to the production of surface-active compounds or enhanced cell aggregation mechanisms [74]. Meanwhile, the limited biofilm-forming ability may be attributed to reduced expression of adhesins or insufficient extracellular matrix production [75]. Notably, the variation observed within *D. hansenii* strains indicates strain-level heterogeneity in biofilm-forming potential [76].

## 4. Conclusions

During this study, cultivable fungal microorganisms associated with honey bee drones were characterized. Eighty-six yeast isolates were distinguished from different developmental stages of honey bee drones collected at the ecological apiary in Lithuania. Yeasts belonging to the genus *Metschnikowia* were detected across all developmental stages of honey bee drones. The highest yeast diversity was observed in sealed larvae, which hosted multiple species, including *M. pulcherrima*, *M. reukaufii*, *M. fructicola*, *S. magnoliae*, *S. srobosivorans*, *S. apis*, *S. apicola*, *S. bombi*, *D. hansenii*, *Z. rouxii*, and *Sporobolomyces* sp. The pupal stage was dominated by *M. pulcherrima*, *S. bombi*, *S. apicola*, *S. magnoliae*, and *D. hansenii*, while adult drones were primarily associated with *M. pulcherrima* and *S. magnoliae*. In the unsealed larvae stage, only *M. pulcherrima* was identified. The analyses of biocidal and probiotic activities showed that the most potent biocontrol ability, along with moderate autoaggregation and hydrophobicity capacity, was exhibited by the yeast species *M. pulcherrima* and *M. fructicola*. Hydrophobicity varied among yeast strains depending on the solvent used in the assay. *S. apis* demonstrated the highest adherence to hexane, while *M. reukaufii* and *S. sorbosivorans* demonstrated the highest adherence to xylene. The strongest ability to form biofilms was observed in *Starmerella* genus yeasts. Microorganisms with pronounced biocidal and probiotic properties contribute significantly to honey bee health by suppressing infections and supporting a balanced gut microbiota. Their presence may enhance immune function, improve nutrient assimilation, and increase resilience to environmental stressors. These beneficial microbes play a vital role in maintaining colony stability and overall bee vitality.

## Figures and Tables

**Table 1:** Assessment of biofilm values at 570 nm. ODc—cut-off value, OD—sample.

OD	Result
OD ≤ ODc	non-biofilm
ODc < OD < 2 × ODc	weak biofilm
2 × ODc < OD < 4 × ODc	moderate biofilm
OD ≥ 4 × ODc	strong biofilm

**Table 2:** Total aerobic counts (TAC) during the processing of different stages of honey bee drones. Numbers (in log CFU/g) are expressed as means ± standard deviation.

Sample	Total Aerobic Counts (TAC), Log (CFU/g)	
	PCA	YPD
Unsealed larvae	4.13 ± 0.54	1.52 ± 0.79
Sealed larvae	2.11 ± 0.37	2.83 ± 0.95
Pupae	3.81 ± 0.84	2.26 ± 0.26
Adults	5.10 ± 0.46	4.60 ± 0.35

**Table 3:** Identification of yeasts isolated from different stages of honey bee drone (ULD—unsealed larvae; SLD—sealed larvae; PD—pupae; AD—adults).

Yeast Species	Strain	Accession No.	Reference Accession No.	Identity (%)
*Metschnikowia pulcherrima*	SLD25	PX353702	OQ305009.1	100
	SLD20	PX353703	OQ304824.1	100
	PD2	PX353704	OR475100.1	99.18
	PD5	PX353705	OR475100.1	100
	AD1	PX353706	OR475100.1	99.79
	AD6	PX353707	MT821086.1	100
	ULD4	PX353708	ON428299.1	99.79
	ULD5	PX353709	ON428298.1	99.58
*Metschnikowia fructicola*	SLD11	PX353710	MZ185370.1	98.56
	SLD39	PX353711	MZ185363.1	99.59
*Starmerella magnoliae*	SLD46	PX353712	NG_060814.1	98.88
	SLD33	PX353713	MH910971.1	99.1
	PD13	PX353714	NG_060814.1	99.55
	PD14	PX353715	NG_060814.1	99.33
	AD3	PX353716	NG_060814.1	98.88
	AD8	PX353717	MH910969.1	99.54
*Metschnikowia reukaufii*	SLD4	PX353718	KM281731.1	100
	SLD2	PX353719	MT472086.1	100
*Starmerella apis*	SLD9	PX353720	PP235714.1	99.77
*Starmerella apicola*	SLD10	PX353721	JN004197.1	99.79
	SLD14	PX353722	KT718105.1	99.17
	PD1	PX353723	KT718105.1	99.38
*Starmerella sorbosivorans*	SLD7	PX353724	MW587726.1	99.88
*Starmerella bombi*	SLD12	PX353726	KY106366.1	99.38
	PD7	PX353727	KY106366.1	99.38
	PD6	PX353728	KY106366.1	100
*Debaryomyces hansenii*	SLD38	PX353729	KY511956.1	100
	SLD42	PX353730	KY511819.1	100
	PD16	PX353731	HM988696.1	100
	PD17	PX353732	KY512083.1	100
*Zygosaccharomyces rouxii*	SLD44	PX353733	KJ739844.1	100
*Sporobolomyces* sp.	SLD48	PX353734	MG588966.1	98.58

**Table 4:** Biofilm-forming ability by yeast isolates from honey bee drone larvae after 72 h.

No.	Strain	OD Average Values ± ST	Biofilm-Forming Ability
1	YPD medium	0.28 ± 0.07	control, non-biofilm
2	*S. cerevisiae flo11*	0.68 ± 0.09	weak biofilm
3	*D. hansenii* SLD38	0.56 ± 0.03	weak biofilm
4	*Z. rouxii* SLD44	0.88 ± 0.13	weak biofilm
5	*M. fructicola* SLD39	1.13 ± 0.22	moderate biofilm
6	*M. reukaufii* SLD2	1.13 ± 0.24	moderate biofilm
7	*M. fructicola* SLD11	1.21 ± 0.05	moderate biofilm
8	*M. pulcherrima* SLD25	1.31 ± 0.70	moderate biofilm
9	*Sporobolomyces* sp. SLD48	1.34 ± 0.12	moderate biofilm
10	*M. pulcherrima* SLD20	1.61 ± 0.18	moderate biofilm
11	*M. reukaufii* SLD4	1.70 ± 0.28	moderate biofilm
12	*D. hansenii* SLD42	2.00 ± 0.30	strong biofilm
13	*S. sorbosivorans* SLD7	2.41 ± 0.07	strong biofilm
14	*S. magnoliae* SLD46	2.58 ± 0.59	strong biofilm
15	*S. apicola* SLD10	2.68 ± 0.48	strong biofilm
16	*S. bombi* SLD12	2.99 ± 0.29	strong biofilm
17	*S. apis* SLD9	3.21 ± 0.38	strong biofilm
18	*S. magnoliae* SLD33	3.56 ± 0.08	strong biofilm
19	*S. apicola* SLD14	3.63 ± 0.01	strong biofilm

## Data Availability

The original contributions presented in this study are included in the article/Supplementary material. Further inquiries can be directed to the corresponding author.

## Supplementary Materials

The following supporting information can be downloaded at: https://www.mdpi.com/article/10.3390/microorganisms13112614/s1, Figure S1: Phylogenetic tree of species inferred from partial sequences of the D1/D2 region (26 rRNA); Table S1: Morphological and molecular analysis of cultivable yeasts isolated from different stages of honey bee drones.
