# Supplementary material for: Diversity and Functional Potential of Yeasts Inhabiting Honey Bee Drones
Source: Microorganisms. 2025 Nov 17;13(11):2614. doi: 10.3390/microorganisms13112614 (PMC12654413; doi:10.3390/microorganisms13112614)
Supplement: Supplementary file 1 [file microorganisms-13-02614-s001.zip › Table S1.pdf]

Table S1. Morphological and molecular analysis of cultivable yeasts isolated from different stages of honey bee drones.

Developmental stage of honey bee drones	Genus	Morphological analysis		PCR product	Profiles of 5.8S-ITS-RFLP analysis (bp)		Analyzed strains
		Colony	Cells		*Hinf*I	*Hha*I	
Unsealed larvae	*Metschnikowia* spp.	Semi-glistening, white to tannish-white, butyrous, low-convex, secreting pulcherrimin	Spherical to ovoid, and occur singly, in pairs, and in small clusters, budding is multilateral	380	200, 180	200, 90, 90	3
Sealed larvae	*Metschnikowia* spp.	Semi-glistening, white to tannish-white, butyrous, low-convex, secreting pulcherrimin	Spherical to ovoid, and occur singly, in pairs, and in small clusters, budding is multilateral	380	200, 180	200, 90, 90	23
	Starmerella spp.	White, convex, smooth, opalescent, butyrous	Ovoid to elongate, and occur singly and in pairs, small	430	230, 200	190, 180, 50	11
				400	250, 120, 120	190, 110, 100	5
				390	200, 190	190, 180, 40	1
	Debaryomyces sp.	Round, flat, white to cream-coloured	Large, oval-shaped, multilateral budding	650	350, 300	300, 280, 70	4
	Zygosaccharomyces sp.	Yellowish creamy to white, matt	Oval to ellipsoidal; budding is multilateral	700	300, 300, 100	330, 180, 150, 50	1
	Sporobolomyces sp.	Distinctive pink to red, round, smooth, glistening	Large, elongated, elliptical	650	280, 140, 120, 110	310, 230, 100	1
Pupae	*Metschnikowia* spp.	Semi-glistening, white to tannish-white, butyrous, low-convex, secreting pulcherrimin	Spherical to ovoid, and occur singly, in pairs, and in small clusters, budding is multilateral	380	200, 180	200, 90, 90	10
	Starmerella spp.	White, convex, smooth, opalescent, butyrous	Ovoid to elongate, and occur singly and in pairs, small	430	230, 200	190, 180, 50	5
				400	250, 120, 120	190, 110, 100	2
				390	200, 190	190, 180, 40	4
	Debaryomyces sp.	Round, flat, white to cream-coloured	Large, oval-shaped, multilateral budding	650	350, 300	300, 280, 70	5
Adults	*Metschnikowia* spp.	Semi-glistening, white to tannish-white, butyrous, low-convex, secreting pulcherrimin	Spherical to ovoid, and occur singly, in pairs, and in small clusters, budding is multilateral	380	200, 180	200, 90, 90	3
	Starmerella spp.	White, convex, smooth, opalescent, butyrous	Ovoid to elongate, and occur singly and in pairs, small	430	230, 200	190, 180, 50	8
