# Supplementary figures and images for: Diversity and Functional Potential of Yeasts Inhabiting Honey Bee Drones
Source: Microorganisms. 2025 Nov 17;13(11):2614. doi: 10.3390/microorganisms13112614 (PMC12654413; doi:10.3390/microorganisms13112614)

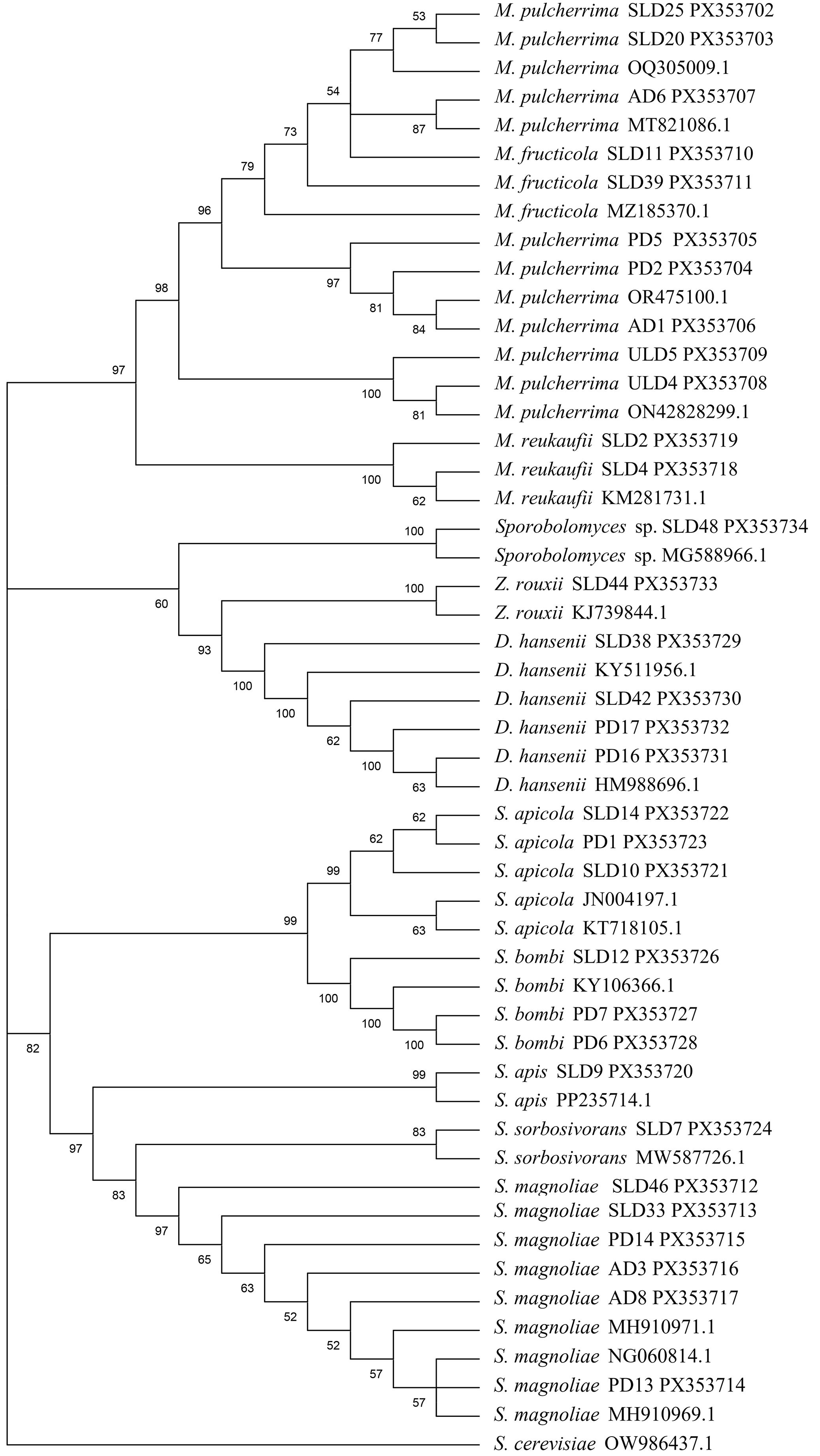

Supplement: Supplementary file 1 [file microorganisms-13-02614-s001.zip › Figure S1.tif]
